# LUNG FUNCTION AND VENTILATORY RESPONSE DURING HIGH-INTENSITY TREADMILL WALKING IN ADULTS WITH CEREBRAL PALSY: A CROSS-SECTIONAL STUDY

**DOI:** 10.2340/jrm.v58.44817

**Published:** 2026-07-23

**Authors:** Eivind LUNDGAARD, Hanna KRAGGERUD, Charlotta HAMRE, Matthijs WOUDA

**Affiliations:** 1 Department of Rehabilitation Science and Health Technology, Faculty of Health Sciences, OsloMet – Oslo Metropolitan University, Oslo; 2 Centre of Research and Education, Sunnaas Rehabilitation Hospital, Bjørnemyr, Norway

**Keywords:** adult, cerebral palsy, physical exertion, spirometry, ventilatory limitation

## Abstract

**Objective:**

To investigate lung function and ventilatory response during cardiopulmonary exercise testing in adults with cerebral palsy and assess the relationship between perceived breathlessness and breathing reserve at maximal exertion.

**Design:**

Prospective, cross-sectional study. Inclusion period October 2023–June 2024.

**Subjects:**

Adults with spastic cerebral palsy, Gross Motor Function Classification Score (GMFCS) I–III (*n* = 100).

**Methods:**

Spirometry and cardiopulmonary exercise testing were conducted. Ventilatory response during exercise was assessed via tidal volume and respiratory rate. Perceived breathlessness was compared with breathing reserve at maximal exertion.

**Results:**

89 participants completed all tests (GMFCS I = 62, GMFCS II = 20, GMFCS III = 7); 90% had lung function within normal limits. Age- and sex-adjusted maximal oxygen uptake was lowest in group III (*p* < 0.01). At maximal exertion, 70% had breathing reserve < 20%, indicating ventilatory limitation. Breathing reserve did not significantly differ between those who did and did not report breathlessness as the limiting factor at maximal exertion. Tidal volume and respiratory rate showed expected values at maximal exertion.

**Conclusion:**

Lung function was normal in most participants, yet ventilatory limitations were common during maximal exercise. Perceived breathlessness was not related to breathing reserve at maximal exertion.

## LAY ABSTRACT

This study explored how adults with cerebral palsy breathe during exercise and how their feelings of breathlessness relate to actual breathing capacity. In total, 89 out of 100 included participants performed lung function and exercise tests according to standardized guidelines. Most participants had normal lung function, but those with more severe mobility challenges had lower breathing capacity and fitness levels. During intense exercise, 70% showed signs of limited breathing reserve, yet only 40% said breathlessness stopped them when performing exercise testing. Interestingly, their actual breathing reserve did not differ much whether they felt breathless or not. Breathing patterns during exercise were typical across all mobility levels. These findings suggest that while lung function is generally normal in adults with cerebral palsy, breathing capacity may still limit the exercise ability.

Cerebral palsy (CP) is a lifelong, non‑degenerative neurodevelopmental condition caused by early brain maldevelopment or injury, affecting muscle tone and motor function that affect daily functioning (1, 2). The Gross Motor Function Classification Scale (GMFCS) classifies motor function, ranging from level I (independent in daily activities) to V (severely affected, totally dependent in daily activities) (3). Across all levels, individuals experience low physical fitness, and significant physical strain of walking (4–6). As they age, many face a decline in walking ability and increased fatigue in which reduced physical fitness has been cited as a contributing factor (7).

For clinical decision-making, it is important to understand the physiological mechanisms leading to changes in physical function and fitness. Maximal oxygen uptake (VO_2_max) is the gold standard for physical fitness (8) and is measured through cardiopulmonary exercise testing (CPET), which evaluates cardiovascular and respiratory function (9). In healthy individuals, cardiac output limits VO_2_max, while the respiratory system is rarely a limiting factor. However, the respiratory system can be a significant constraint in individuals with pulmonary disease, the elderly, and athletes (9–11). The impact of lung function on VO_2_max is often assessed by measuring the difference between ventilatory capacity, measured as maximal voluntary ventilation at rest, and maximal ventilation during exercise testing, expressed as breathing reserve (BR) (9). If ventilation exceeds a certain percentage of the BR, ventilatory limitation during physical exertion is indicated. The threshold for ventilatory limitation varies in the literature with BR cut-offs from 15–40% (9). A BR of 20% was chosen as a cut-off for ventilatory limitation in this study.

Despite its potential impact on physical fitness, lung function in adults with CP has been poorly studied. There is evidence that CP may impair respiratory function, and respiratory disease has been identified as the leading cause of mortality in individuals with CP (12). One notable study by Lampe and colleagues found reduced lung function in adults with CP, with a negative correlation between GMFCS level and lung function (13). It is thus plausible that lung function might be a limiting factor during exercise in adults with CP. To the authors’ knowledge there are no studies reporting ventilatory parameters during exercise in adults with CP.

The staff at the clinical physiological laboratory at Sunnaas Rehabilitation Hospital have extensive experience with CPET in adults with CP. Over the years individuals with CP have reported breathlessness as the primary limitation in their exercise tolerance, most frequently in those with severe CP. Whether this is caused by obstructive and/or restrictive lung function, decreased ventilatory efficiency, or related to low physical fitness remains unanswered and requires further investigation.

To address these knowledge gaps, we conducted spirometry and CPET in a cohort of adults with CP GMFCS level I–III. The study had 3 objectives. First, we compared lung function across GMFCS levels, hypothesizing that lung function was associated with the severity of CP. Second, we explored the ventilatory response during CPET. We hypothesized that severity of CP was associated with a smaller tidal volume increase and higher respiratory rate during exertion. Finally, we compared subjective reports of ventilatory limitation with objective measurements of BR at maximal exertion. We hypothesized lower BR in those reporting ventilatory constraints during maximal exertion.

## METHODS

### Design and setting

This cross-sectional study is a part of the “Cost of Walking in Adults with Cerebral palsy” (COWAC) study (14) at Sunnaas Rehabilitation Hospital (SRH) in Norway.

### Ethical approval

The study was approved by the Regional Committee for Medical and Health Research Ethics, South-East Norway (REK-number 530205), and data handling approval was obtained from the Norwegian Agency for Shard Services in Education and Research (SIKT-number 655023) before data collection. The study was conducted in accordance with the laws and regulations in force in Norway.

### Recruitment, inclusion and exclusion criteria

Participants were recruited between September 2023 and June 2024, through 3 primary sources: (*i*) inpatients at SRH, (*ii*) public announcements posted on the website of the Norwegian Cerebral Palsy Association, and (*iii*) individuals who had previously participated in the study conducted by Maanum et al. (15) between 2008 and 2011, and took part in a 16-year follow-up study (16). As data presented in this paper originate from a larger study (16), no separate sample size calculation was performed for the recent sub-study. For the same reason, recruitment was not designed to achieve equal numbers of participants across the 3 GMFCS groups.

The inclusion criteria were individuals with unilateral or bilateral spastic CP, age 18–65 years, GMFCS level I, II, or III, able to walk continuously for 5 min, with or without a walking aid, and having had normal schooling. Exclusion criteria were known cardiovascular or pulmonary diseases that could affect exercise tolerance or pose a safety risk, orthopaedic surgery within the last 18 months, injections with spasticity-reducing medication within the last 3 months, or pregnancy. Potential participants were given oral and written information concerning the study and confirmed their participation by signing consent papers.

### Procedures

After inclusion, participants had a physical examination by an experienced physiotherapist at SRH to ensure that they were suitable to undergo the test protocol. During this examination demographic and medical data were collected (e.g., age, sex, spasticity, uni- or bilateral, GMFCS level, smoking status, asthma/blood pressure medicine use). Height and weight were objectively measured. Body mass index (BMI) was calculated with the formula weight (kg)/height (metres)^2^. The physical examination, spirometry, and CPET, including measurement of oxygen cost of walking (O_2_-cost), were performed in that order at the clinical physiological laboratory at SRH by the same physiotherapist.

### Spirometry

Lung function was measured in a sitting position with a Quark CPET (COSMED, Albani Laziale, Rome, Italy) according to the 2019 updated ATS guidelines (17). Maximal voluntary ventilation (MVV) was performed in a 12 s maximal ventilation manoeuvre. The Global Lung function Initiative network (GLI) and European Respiration Society (ERS) have collected normative data and developed reference equations that facilitate standardized interpretation of lung function results and which were used (18, 19).

The outcome measures for the spirometry were forced vital capacity (FVC), forced expiratory volume in 1 s (FEV1), the ratio between FEV1 and FVC (FEV%), peak expiratory flow (PEF), and MVV, measured and calculated (see data analysis). Lower limits of normal (LLN) was defined as the fifth percentile corresponding to a z-score of –1.645.

### Oxygen cost of walking and cardiopulmonary exercise test

The participants performed a test of O_2_ cost, which is an indicator of the walking efficiency (20). Prior to the O_2_ cost test, the participants’ self-selected walking speed (SSWS) was determined by modifying the procedure described by Martin et al. (21). The participants walked on a treadmill (PPS med, Woodway, Waukesha, WI, USA), starting at 0.3 m∙s^–1^. Speed was then slowly increased until SSWS was identified and adjusted according to the participants’ perception. SSWS was maintained for 1 min, before the treadmill speed was gradually increased up to their individual maximum walking speed. Further, speed was decreased to the identified SSWS and adjusted again according to the participants’ perception. The sequence lasted for about 5 min. After a 5-min rest, O_2_ cost was measured over a 5-min period at the participant’s SSWS.

After a 10-min rest, the participants performed a CPET with measurement of VO_2_max (8). This was done following the Modified Balke Protocol (22). The participants walked on the treadmill at their SSWS with a gradual increase in incline, 2% per minute (starting at 2%), until voluntary exhaustion. If the incline reached 20%, the speed was increased by 10% of SSWS each minute. Participants were instructed to exercise to their maximal intensity, with vigorous verbal encouragement expressed by the physiotherapist. Due to walking impairments and safety concerns during treadmill testing, all participants were permitted to hold onto the handrail of the treadmill with 1 or 2 hands with light weightbearing if necessary. Reasons for determination of the CPET were defined as breathlessness, leg discomfort, general exhaustion, balance problems, and pain.

Throughout these 2 tests, oxygen uptake (VO_2_ ml∙min^–1^), carbon dioxide output (VCO_2_ ml∙min^–1^), minute ventilation (VE; L∙min^–1^), tidal volume (VT; L), and respiratory rate (RR; breaths per minute) were measured continuously.

Parameters were measured using a computerized standard open-circuit technique breath-by-breath spirometer (Quark CPET, COSMED, Albani Laziale, Rome, Italy).

Heart rate was measured throughout the treadmill testing using a 3-lead ECG monitor (Tango M2, SunTech Medical, Morrisville, NC, USA). Capillary blood was taken from a finger immediately after the O_2_ cost test and 2–3 minutes after the CPET to measure blood lactate (Biosen C-Line EKF-diagnostic, Barleben, Germany).

### Calibration

The spirometer was calibrated before each test. A 3-L calibration syringe was used for volume calibration. For calibrating the gas analysers, medically certified calibration gases (16% O_2_/5% CO_2_) and room air were used. The Biosen lactate analyser was calibrated with a 12.00 mmol lactate L^–1^ solution prior to each blood sample.

### Data analysis

MVV was calculated with the formulas FEV1*35 and FEV1*40 (9). Gas exchange values were time-averaged into 30-s intervals. The highest 30 s interval were considered VO_2_max. Values from the fourth and fifth minute of the O_2_ cost test were used to calculate O_2_ cost of walking (VO_2_ [ml∙kg^–1^∙min^–1^]/walking speed [m∙min^–1^] = VO_2_ ml∙kg^–1^∙m^–1^). To calculate the participants’ ventilation at maximal exertion as a percentage of their own MVV (VE%MVV), the formula VE/MVV ×100 was used. BR was calculated using the formula MVV-VE (23). A BR < 20% was defined as ventilatory limitation. BR was dichotomized into preserved (≥ 20%) and reduced (< 20%) (24). Ventilatory efficiency, expressed as VE/VCO₂ nadir, was determined as the lowest mean value derived from 3 consecutive 30‑s averaged VE/VCO₂ intervals, as described by Sun et al. (25).

### Evaluation of the cardiopulmonary exercise test

To evaluate the CPET, some criteria had to be met: for respiratory exchange ratio (RER) and blood lactate (La^–1^) we used age- and sex-related reference values (26). Other criteria were a VO_2_ plateau (⩽ 2 ml∙kg^–1^∙min^−1^ rise in VO_2_ despite increased workload at maximal intensity) and ≥ 90% predicted maximal heart rate (HR max), using the formula (211–[0.64*age]) (27, 28).

VO_2_max reference values presented in the large Norwegian study by Edvardsen et al. were used (29). Physical fitness was defined as reduced when VO_2_max < 85% of predicted (9). Ventilatory equivalent reference values from Sun et al. and Loe et al. were used (25, 30).

### Statistics

All statistical analysis were performed using the Statistical Package for the Social Sciences (SPSS) version 27 (IBM Corp, Armonk, N, USAY) program. Normality was assessed using visual inspection of histograms, supplemented by the Shapiro–Wilk test. Continuous variables are presented with median and interquartile range (IQR). Categorical variables are presented with absolute numbers and percentages. Difference in outcome measures between GMFCS levels was tested with the Kruskal–Wallis test. When significant differences were detected by Kruskal–Wallis test, the Mann–Whitney *U* test was used for *post hoc* analysis. A *p*-value < 0.05 was regarded as statistically significant. To investigate the association between lung function and exercise variables Spearman’s correlation coefficient was used and interpreted according to Schober et al. (31). Simple scatterplots were used to illustrate correlations. The χ^2^ test was used to examine the relationship between breathing reserve and symptoms of breathlessness.

## RESULTS

Non‑parametric tests were used because several variables were non‑normally distributed, and the number of participants differed across the 3 GMFCS groups.

### Participants

A total of 100 adults with CP were recruited to this study. Eighty-nine completed the test procedure and were included in the analysis. Demographics and health-related data are presented in Table I. Seven participants were pre-diagnosed with asthma; 3 of them had an obstructive lung function measured by spirometry. Five participants (3 regularly) used inhalants (short-acting beta-agonist) and 7 were ever smokers. None were diagnosed with chronic obstructive pulmonary disease (COPD). Eleven participants were excluded from data analysis, as they did not perform spirometry and/or CPET according to standardized guidelines. The excluded participants were classified by the GMFCS at level I (*n* = 3), level II (*n* = 6), and level III (*n* = 2), 3 with uni- and 8 with bilateral spastic CP. No incidents of adverse events were reported during the procedure.

**Table I:** Participant characteristics

Item	GMFCS I	GMFCS II	GMFCS III	Total
*n* (%)	62 (70)	20 (22)	7 (8)	89 (100)
Age, years	39 (31, 47)	41 (30, 51)	55 (42, 68)	40 (32, 49)
Sex (female), *n* (%)	35 (57)	10 (50)	5 (71)	50 (56)
Height, cm	172 (164, 180)	171 (167, 176)	163 (153, 173)	170 (163, 178)
Weight, kg	74 (63, 85)	73 (61, 86)	70 (63, 78)	73 (62, 83)
BMI, kg/m^2^	25 (22, 28)	26 (24, 29)	27 (25, 29)	26 (23, 29)
Hemiplegia, *n* (%)	50 (81)	7 (35)	0	57 (64)
Diplegia, *n* (%)	12 (19)	13 (65)	7 (100)	32 (36)
Baclofen, *n* (%)	3 (5)	1 (5)	2 (29)	6 (7)
Asthma, *n* (%)	5 (8)	2 (10)	0	7 (8)

Values are presented as median (IQR) or *n* (%). IQR: interquartile range; GMFCS: Gross Motor Function Classification System; BMI: body mass index.

### Resting lung function

Table II gives an overview of the results from the spirometry. About 90% of the participants had lung function within normal values, i.e., above LLN for healthy individuals. When comparing lung function values between the different GMFCS levels, there was no statistically significant difference in percentage expected in FVC, FEV1, PEF, and MVV. In total, 8 participants, including 1 ever smoker, were defined with obstructive lung function (FEV% z-score < –1.64), graded mild to moderate. The lowest FEV1 was 68% of expected and the lowest FEV% was 62%.

**Table II:** Spirometry

Item	GMFCS I	GMFCS II	GMFCS III	Total	*p*-value
FVC, litres	4.19 (3.33, 5.05)	4.34 (3.75, 4.93)	3.2 (2.5, 3.9)	4.21 (3.42, 5.01)	0.10
FVC%	100 (94, 107)	106 (95, 116)	106 (95, 117)	101 (94, 108)	0.20
FVC ≥ LLN, *n* (%)	56 (90)	19 (95)	7 (100)	82 (92)	
FEV1, litres	3.42 (2.92,3.92)	3.35 (2.69, 4.02)	2.7 (2.00, 3.41)	3.36 (2.81, 3.92)	0.19
FEV1%	99 (90, 109)	105 (97, 113)	104 (93, 115)	101 (92, 110)	0.44
FEV1 ≥ LLN, *n* (%)	57 (92)	18 (90)	6 (86)	81 (91)	
FEV% #	82 (79, 86)	79 (77, 82)	82 (78, 87)	81 (78, 85)	0.09
FEV% ≥ LLN, *n* (%)	57 (92)	18 (90)	7 (100)	82 (92)	
PEF, litre∙min	490 (398, 582)	442 (321, 564)	407 (364, 451)	475 (376, 575)	0.29
PEF%	106 (91, 120)	109 (93, 125)	110 (97, 123)	109 (95, 123)	0.74
MVV, litre∙min	124 (103, 145)	121 (99, 142)	94 (84, 105)	121 (100, 142)	0.03*
MVV%	99 (90, 107)	100 (85, 114)	88 (79, 97)	98 (88, 108)	0.33
MVV calculated FEV1x35, l∙min	120 (101, 138)	118 (97, 139)	95 (70, 119)	118 (98, 138)	0.18
MVV calculated FEV1x40, l∙min	137 (116, 158)	134 (110, 158)	108 (80, 136)	134 (112, 157)	0.18

Values are presented as median (IQR) or *n* (%). FVC: forced vital capacity; LLN: lower limit of normal; FEV1: forced expiratory volume 1. second. FEV: forced expiratory volume; PEF: peak expiratory flow; MVV: maximal voluntary ventilation. #FEV% = (FEV1/FVC) x100.

* GMFCS III is statistically significantly different from GMFCS I and II (*p* < 0.05).

### Heart rate and oxygen consumption during walking at submaximal and maximal exercise intensity

Table III presents the results of the O_2_ cost and CPET. The SSWS was highest in participants with GMFCS I. Expressed as percentage of expected; VO_2_ max (ml∙kg^–1^∙min^–1^) was significantly higher in GMFCS I compared with II and III (*p* < 0.01). In total, 60 participants had a VO_2_ max > 85% of expected: GMFCS I (*n* = 46), GMFCS II (*n* = 12), and GMFCS III (*n* = 2). Physiological parameters (% VO_2_max, HR, blood lactate, ventilation) at SSWS were significantly higher in GMFCS III compared with GMFCS I and II (*p* < 0.001) and higher in GMFCS II compared with GMFCS I (*p* < 0.001). The participants reached a high level of exhaustion when tested for maximal oxygen uptake. Eighty-seven subjects achieved at least 2 criteria for VO_2_max and were thus considered at maximum effort. Breathlessness was the main reason for termination of the exercise test in 36 subjects (40%), while 40 subjects (45%) experienced leg discomfort. A minor number of the sample experienced exhaustion (6%) or balance problems (6%) as the main reason for terminating the test.

**Table III:** Heart rate and oxygen consumption at self-selected walking speed and maximal exercise test

Item	GMFCS I	GMFCS II	GMFCS III	Total	*p*-value
HR SSWS, strokes∙min^–1^	110 (100, 121)	122 (108, 136)	136 (123, 149)	118 (105, 131)	< 0.001#*
VO_2_ ml∙min^–1^ SSWS	1235 (1037, 1434)	1477 (1169, 1785)	1282 (1109, 1455)	1256 (1022, 1489)	0.455
SSWS, m∙s^–1^	1.23 (1.13, 1.33)	0.91 (0.76, 1.05)	0.5 (0.29, 0.71)	1.12 (0.96, 1.28)	< 0.001#*
% of VO_2_ max SSWS	45 (39, 51)	56 (49, 63)	81 (75, 87)	50 (41, 58)	< 0.001#*
% of HR max SSWS	61 (56, 67)	69 (62, 76)	87 (79, 96)	64 (57, 71)	< 0.001#*
Lactate mmol^–1^ SSWS	0.94 (0.69, 1.19)	1.27 (0.92, 1.62)	3.69 (2.94, 4.45)	1.04 (0.75, 1.34)	< 0.001#*
HR max, strokes∙min^–1^	183 (176, 191)	184 (174, 194)	173 (160, 186)	182 (174, 190)	0.159
HR max % of age predicted	99 (95, 103)	100 (94, 106)	97 (93, 102)	99 (95, 103)	0.714
VO_2_ max ml∙min^–1^	2642 (2140, 3145)	2745 (2313, 3178)	1650 (1434, 1866)	2623 (2108, 3139)	0.002*
VO_2_ max ml∙min^–1^ % of expected	97 (85, 110)	90 (75, 106)	77 (65, 90)	91 (78, 105)	0.012#*
VO_2_ max ml∙kg^–1^∙min^–1^	37.0 (32.0, 42.1)	32.3 (27.1, 37.6)	25.1 (21.2, 29.0)	35.8 (29.4, 42,2)	< 0.001#*
VO_2_ max ml∙kg^–1^∙min^–1^ % of expected	96 (81, 110)	89 (79, 100)	81 (65, 98)	94 (81, 106)	0.002#*
Walking speed maximal exertion, m∙s^–1^	1.33 (1.18, 1.48)	0.97 (0.85, 1.09)	0.51 (0.44, 0.58)	1.17 (0.98, 1,36)	< 0.001#*
Incline, maximal exertion, %^a^	20 (12, 20)	20 (16, 20)	16 (14, 20)	20 (12, 20)	0.005#
Lactate mmol^–1^ max	9.99 (8.00, 11.97)	8.81 (6.50, 11.13)	7.94 (6.46, 9.43)	9.11 (7.19, 11.02)	0.038#*
RER max	1.10 (1.05, 1.15)	1.04 (0.99, 1.09)	1.01 (0.98, 1.05)	1.07 (1.01, 1.13)	< 0.001#

Values are presented as median (IQR).

a Values presented as median (min, max). GMFCS: Gross Motor Function Classification Score; HR: heart rate; SSWS: self-selected walking speed; VO_2_: oxygen consumption; VO_2_ max: maximal oxygen uptake; HR max: maximal heart rate; RER: respiratory exchange ratio.

# GMFCS I is significantly different from GMFCS II and III.

* GMFCS I and II are significantly different from GMFCS III (*p*< 0.05).

### Ventilation at submaximal (SSWS) and maximal exercise intensity

**Fig. 1:**
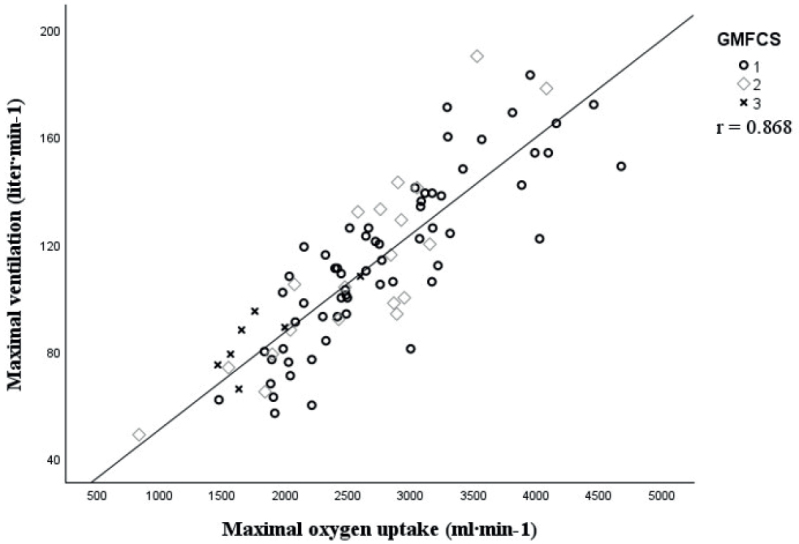
Maximal ventilation and maximal oxygen uptake at maximal exercise intensity.

The participants’ tidal volume at maximal intensity (VT max) was on average 2.17 L, RR 52 breaths/min and maximal ventilation (VEmax) 108 L∙min^–1^ (Table IV). The median VE max was 89% of MVV and thus yielding a BR of 11%. VEmax was higher in participants with GMFCS I and II compared with level III and showed a high correlation with VO_2_max (*r* = 0.868) (Fig. 1). For MVV measured during regular spirometry, we found a positive correlation with VEmax (*r* = 0.740). There was no difference in maximal RR or VT as a percentage of FVC (VTmax % FVC) between GMFCS levels. Ventilatory equivalents were elevated across all GMFCS levels (Table IV).

**Table IV:** Ventilation parameters at self-selected walking speed and maximal intensity

Item	GMFCS I	GMFCS II	GMFCS III	Total	*p*-value
VT SSWS, litres	1.32 (1.11, 1.53)	1.35 (1.20, 1.51)	1.42 (1.18, 1.67)	1.34 (1.14, 1.53)	0.66
VT SSWS % of FVC	30 (25, 35)	30 (26, 35)	41 (32, 51)	30 (25, 35)	0.15
RR SSWS, breaths∙min^–1^	27 (24, 31)	28 (22, 34)	32 (24, 41)	27 (23, 32)	0.01*
VE SSWS, litre∙min^–1^	33 (28, 39)	39 (28, 51)	47 (35, 60)	36 (29, 43)	< 0.01*
VE SSWS % of MVV	26 (20, 33)	32 (25, 39)	49 (43, 56)	29 (23, 36)	< 0.001*
VT max, litres	2.29 (1.95, 2.63)	2.14 (1.78, 2.49)	1.71 (1.50, 1.92)	2.17 (1.83, 2.50)	0.01*
VT max % of FVC	53 (48, 58)	51 (43, 58)	51 (40, 63)	53 (47, 58)	0.24
RR max, breaths∙min^–1^	51 (44, 58)	51 (43, 59)	52 (48, 56)	52 (45, 59)	0.86
VE max, litre∙min^–1^	111 (89, 134)	104 (81, 128)	88 (78, 98)	108 (86, 131)	0.04*
VE max % of MVV	88 (78, 99)	89 (71, 107)	94 (81, 108)	89 (78, 101)	0.98
Ve/VCO_2_ nadir	29.6 (26.9, 32.3)	32.2 (29.3, 35.2)	39.9 (35.4, 44.5)	30.6 (28.0, 33.3)	< 0.001*#
Ve/VCO_2_ nadir, % of expected	111 (104, 119)	122 (113, 131)	140 (133, 146)	115 (105, 126)	< 0.001*#
Highest Ve/VCO_2_	35.5 (32.6, 38.5)	39.2 (34.0, 44.4)	45 (38.9, 51.0)	36.3 (32.8, 39.9)	< 0.001*#
Highest Ve/VCO_2_, % of expected	118 (109, 128)	131 (114, 148)	148 (130, 167)	122 (110, 133)	< 0.001*#
Highest Ve/VO_2_	37.9 (34.4, 41.4)	40.6 (35.9, 45.3)	48.2 (42.7, 53.7)	39.3 (35.2, 43.4)	0.015*
Highest Ve/VO_2_, % of expected	114 (103, 125)	122 (108, 136)	146 (130, 163)	117 (105, 130)	< 0.001*
BR l∙min^–1^	12 (–44, 79)	15 (–34, 87)	4 (–2, 45)	12 (–44, 87)	0.95
BR %	12 (2, 23)	11 (–7, 29)	6 (–8, 20)	11 (–1, 23)	0.97
BR ≥ 20%, *n* (%)	18 (29)	7 (35)	2 (29)	27 (30)	
BR %, MVV calc. £	20 (9,32)	21 (12, 30)	17 (5, 30)	20 (9, 32)	0.56
BR %, ≥ 20%, *n* (%) MVV calc. £	31 (50)	11 (55)	3 (43)	45 (51)	

Values are presented as median (IQR) or *n* (%). GMFCS: Gross Motor Function Classification Score; VT: tidal volume; SSWS: self-selected walking speed; FVC: forced vital capacity; RR: respiratory rate; VE: ventilation; MVV: maximal voluntary ventilation; BR: breathing reserve; FEV1: forced expiratory volume 1. second.

* GMFCS III is significantly different from GMFCS I and II (*p* < 0.05).

# :GMFCS I is significantly different from GMFCS II and III. £: MVV calculated: FEV1x40.

**Fig. 2:**
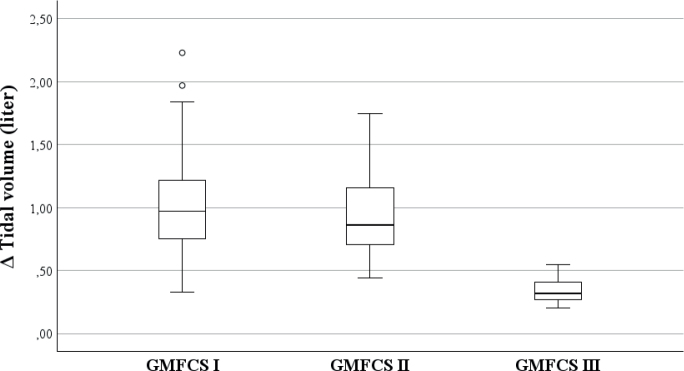
Changes (Δ) in tidal volume from submaximal (self-selected walking speed) to maximal exercise intensity for Gross Motor Function Classification Score (GMFCS) I–III.

There was a significant increase in VT from SSWS to maximal intensity across all GMFCS levels (median [interquartile range] 0.92 litre [0.2, 2.33], *p* < 0.001). The increase was higher in GMFCS I and II than level III (Fig. 2). Participants with GMFCS III had higher ventilation (also as % MVV) and RR on SSWS (*p* < 0.01) compared with GMFCS I and II. The correlation between ventilation and oxygen consumption at SSWS was high (*r* = 0.868).

### Perceived breathlessness and breathing reserve at maximal ventilation

Thirty-six participants (40%) experienced ventilation (breathlessness) as the limiting factor when performing the maximal exercise test. There was no significant difference in either VE max or BR% between those who experienced breathlessness vs those who did not (VEmax median (IQR)) 109 (47) vs 108 (48), *p* > 0.05 and BR% 9 (27) vs 14 (23), *p* > 0.05).

There was an insignificant correlation between GMFCS levels I–III and BR and between lung function variables (FVC, FEV1, MVV) and BR (*r* between 0.200 and –0.200). When calculating BR% using direct measurement of MVV, 62 participants (70%) had a BR of less than 20%, indicating ventilatory limitation at maximal exercise. Twenty-three individuals, 9 females and 14 males, had a higher VEmax than MVV (BR ≤ 0%). The distribution across GMFCS levels and types of CP for those 23 individuals was similar to the rest of the group. Calculating MVV (FEV1*40) revealed BR< 20% in 44 participants (49%). There was a low, negative correlation between BR% and VO_2_ max, *r* = –0.445.

## DISCUSSION

This study examined lung function and ventilatory response during submaximal and maximal treadmill walking in adults with CP. Of the 100 participants recruited, 89 completed all tests by standardized protocols. There was a wide range of variations in lung function and fitness levels, with the majority reaching values above the LLN. Ventilatory response, O_2_ cost, and VO_2_max were associated with the severity of cerebral palsy. Notably, 62 participants had BR below 20%, indicating ventilatory limitation, and 36 reported breathlessness as the primary limiting factor during maximal exercise. The markedly uneven distribution of participants across the GMFCS levels reduced statistical power and limited the reliability of comparative analyses. As a result of these limitations, differences in outcomes across GMFCS levels should be interpreted with caution. The first objective was to compare lung function across GMFCS levels. All lung function variables were within normal range across GMFCS levels. Given the differences in sex distribution, age, and height between the GMFCS levels, these factors may contribute to the observed differences in absolute values. The GLI reference values used in this study have been evaluated against a large, healthy Norwegian reference sample and were found to fit better than other reference values (32). Approximately 90% of our study sample had FVC, FEV1, and FEV% values above LLN. This is slightly lower than the Norwegian reference sample, where 2–3% had values below LLN (32). The same study found a prevalence of obstruction, defined as FEV% < LLN, of 7%, which agrees with a prevalence in obstruction of 8% in our sample.

To the best of our knowledge, 2 studies have reported lung function measured by spirometry in adults with CP. In a study by Lampe et al. (13) comprising 46 adults with CP, GMFCS I–IV measured vital capacity (VC). They documented reduced VC compared with matched reference values at all GMFCS levels. VC decreased with the increase of GMFCS, and GMFCS level I and II had statistically significant higher VC than GMFCS level III. However, Lampe et al. (13) did not report any anthropometric measures or percentage of normative values, which makes comparison with our results difficult. In a recent study by Corell et al. (33), lung function was assessed in a cohort of adults with CP, revealing an average vital capacity of approximately 50% of the predicted value. However, these participants were more severely affected (9 out of 16 participants had GMFCS levels IV–V) and included individuals with spastic, dyskinetic, and ataxic CP. These results are difficult to compare with our results, but suggest that greater severity and specific CP subtypes may be associated with more pronounced reductions in lung function (33).

The second objective was to describe ventilation during SSWS and maximal exercise in adults with CP. We found a wide range of ventilatory responses at both exercise intensity levels. Not surprisingly, VEmax was highly correlated with VO_2_max, as ventilation is primarily driven by metabolic demands (23). Every participant showed a significant increase in both VT and RR from SSWS to maximal exercise. This is consistent with findings in several other studies investigating ventilatory response during exercise in healthy subjects (23, 30, 35). In healthy adults, VT during comparable submaximal exercise corresponds to 25–35% of FVC (36). In the present study, the relative VT at SSWS is largely comparable to this range, although the GMFCS III group operated at a higher proportion of FVC, probably due to higher relative load during walking. At maximal exercise, all groups reached similar relative tidal volumes (≈50% of FVC), consistent with a plateau of 50–60%, seen in a healthy population (23).

Respiratory rate during SSWS (27–32 breaths/min) was within or slightly above the upper range reported for healthy individuals, where respiratory rates typically ranges from 20–30 during comparable exercise intensities (30). Respiratory rate peak values (RR≈50) were higher than 35–45, as has been reported in healthy adults (23, 30). This pattern suggests a preserved ability to increase tidal volume and respiratory rate appropriately with exercise intensity. At SSWS, ventilatory parameters were highest in GMFCS III, reflecting a higher physical load of walking, confirmed by higher blood lactate levels and physical strain (% VO_2_max/% HRmax). An intensity of 81% of VO₂max seen during SSWS in GMFCS III corresponds to high-intensity aerobic training and has the potential to provide a meaningful stimulus for improving aerobic capacity (37). In contrast, individuals with GMFCS level III demonstrated lower VO₂max despite high relative physiological strain, a pattern attributed to generally low levels of physical activity (38). Despite high physical strain, participants with GMFCS level III still exhibited substantial BR of approximately 50% at SSWS, indicating that although walking is physiologically demanding, it does not fully tax their ventilatory system.

**Fig. 3:**
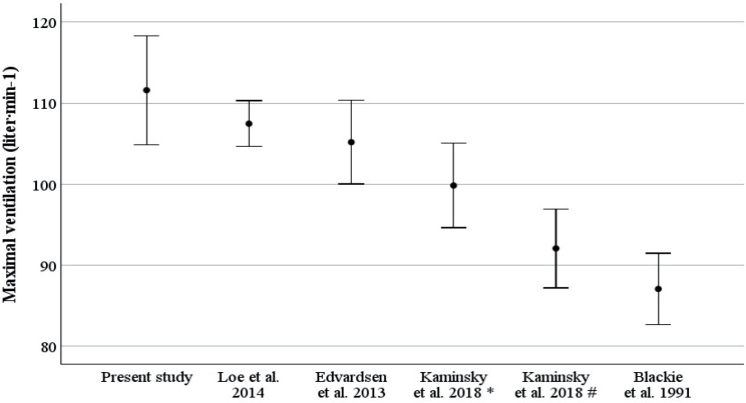
Measured means (95% CI) maximal ventilation for all participants in the present study and estimated values for the same participants based on equations and reference values from other studies. Loe: prediction equation; Ve_peak_ (L∙min^–1^) = 16.843+ (0.178 x weight) + (0.876 x height) – (0.762 x age) – (28.171 x gender) (gender; male = 1, female = 2). Edvardsen: expected maximal ventilation based on age- and sex-specific reference values. Kaminsky*: prediction equation; Ve_peak_ (L∙min^–1^) = 5.51 + 26.79 (FEV1 [L]). Kaminsky#: sex-specific reference values for maximal ventilation, 50th percentile. Blackie: prediction equation: Ve_peak_ (l∙min^–1^) = 12.34 + 28 (VO_2max_ l∙min^–1^) (24, 25, 32, 33).

Our study sample reached higher ventilatory values at exhaustion compared with predicted and several other reference values for maximal ventilation in healthy adults (23, 29, 30, 35), as shown in Fig. 3. These findings are not related to high oxygen uptake, as VO_2_max in our sample was slightly lower than the reference sample. Elevated ventilatory equivalents for CO_2_ at submaximal and maximal exercise intensities indicate ventilatory inefficiency, consistent with findings reported by Corell et al. in cardiopulmonary exercise testing of adults with cerebral palsy (33). Elevated VE/VCO_2_ values are frequently associated with cardiopulmonary disease (39). Nevertheless, many participants in this study exhibited normal lung function and VO_2_ max values within the expected range, indicating preserved cardiopulmonary function. This suggests that factors other than overt cardiac or pulmonary dysfunction may contribute to the elevated VE/VCO_2_ observed.

Finally, we wanted to determine whether adults with CP experienced ventilatory limitations during exercise, and if this was associated with reduced BR. When calculating BR, adults with CP in this study had an average maximum ventilation of 89% of MVV, which indicates that they are ventilatory limited, as maximal ventilation is normally 60–80% of MVV (35). Heterogeneity in the BR among adults with CP in this study was observed, with a large proportion of the participants demonstrating a reduced BR defined as BR < 20%. We found a small, negative correlation between BR% and VO_2_max (*r* = –0.445). This suggests that the physical fitness level cannot explain the degree of ventilatory limitation during maximal exhaustion in adults with CP in this study. The correlation between MVV and BR% was negligible, underscoring the limitation of using MVV as a predictor for ventilatory capacity for adults with CP. It is also consistent with previous research conducted in healthy individuals, which emphasizes the metabolic drive behind ventilation (23, 35).

Our results showed wide ranges of BR, which is supported by the results found by Blackie et al. (23), where wide ranges of measured BR in healthy individuals at maximal exhaustion were found. The same study suggests that the wide range of “normal” responses must be taken into consideration when maximal ventilatory data from exercise tests are analysed. Similarly, our findings underscore the importance of considering individual variability when interpreting BR data in adults with CP. BR could not discriminate between those who experienced ventilatory limitations and those who did not. To our knowledge, no other studies have examined the relationship between subjective ratings of ventilatory limitations and measurement of breathing reserve. Thirty-six participants (40%) experienced ventilation as the main exercise limitation. This corresponds to findings from a study of around 1,000 healthy Norwegian adults performing exercise testing, where 38% of the female participants experienced dyspnoea as the limitation for exercise testing (29). The pulmonary system is often considered over-dimensioned, and a significant BR at maximal exercise is expected despite large variability in ventilatory response (9). Results from our study only partially support this claim. There was, however, no indication of any pathological limitation to exercise in the sample. Reaching an expected HRmax indicates no pathological limitation to exercise (9). As our sample reached 100% of predicted HRmax, no pathological limitation should be considered. Nevertheless, other potential limitations to VO_2_ max should be considered. Cardiac output is a key determinant of VO_2_ max, along with peripheral factors such as oxygen extraction capacity and muscular fatigue (40). Furthermore, impairments in coordination and balance may also have contributed to participants who reported muscular fatigue as their main limiting factor.

### Methodological considerations

The study sample is well characterized, with a distribution of demographic and clinical characteristics that closely align with the broader CP population, strengthening the external validity of the results. The validity of the test results is considered good. All tests were supervised by experienced test personnel and performed according to ATS guidelines (17). Tests that did not meet the requirements of the ATS standard were excluded from the data analysis. There is, however, one concern. Residual lactate from the submaximal workload may have influenced lactate responses to maximal exercise, as the 10‑min recovery period may not have allowed full lactate normalization in participants with GMFCS III.

This study does have limitations. The study sample was not a random sample. Participants who register to participate in fitness studies tend to have better health and thus a higher fitness level (29, 30). We managed to include a high number of participants but the distribution between the GMFCS levels was skewed. A small number of participants with GMFCS III may have led to a lack of statistical power to prove differences between the groups and thereby answer the stated objectives.

Participants demonstrated low BR and high ventilatory equivalents during exertion. The absence of arterial blood gas measurements (PaO_2_ and PCO_2_) limited our ability to assess exercise‑induced hypoxemia and hyperventilation during high ventilatory demand. In addition, the lack of lung diffusion capacity measurements restricted evaluation of gas diffusion as a potential contributor to exercise limitation. Consequently, mechanistic interpretation of ventilatory responses during exercise remains incomplete.

In conclusion, this study provides novel insights into lung function and ventilatory response during exercise in adults with CP. Resting lung function was largely within normal limits across GMFCS levels, with minimal evidence of obstructive patterns. However, ventilatory response during submaximal and maximal treadmill walking revealed substantial heterogeneity, particularly in BR, which was reduced in a significant proportion of participants. Despite reaching predicted maximal heart rates and demonstrating no pathological exercise limitations, 40% of participants reported ventilation as the primary limiting factor during maximal exertion.

The findings suggest that ventilatory limitation in adults with CP is not solely attributable to reduced physical fitness or maximal voluntary ventilation but may reflect inefficiencies in ventilatory control or increased metabolic demand during ambulation. Importantly, BR did not reliably discriminate between those who experienced ventilatory limitation and those who did not.
